# Down-Regulation of Replication Factor C-40 (RFC40) Causes Chromosomal Missegregation in Neonatal and Hypertrophic Adult Rat Cardiac Myocytes

**DOI:** 10.1371/journal.pone.0039009

**Published:** 2012-06-14

**Authors:** Hirotaka Ata, Deepa Shrestha, Masahiko Oka, Rikuo Ochi, Chian Ju Jong, Sarah Gebb, John Benjamin, Stephen Schaffer, Holly H. Hobart, James Downey, Ivan McMurtry, Rakhee Gupte

**Affiliations:** 1 Biochemistry & Molecular Biology, University of South Alabama, Mobile, Alabama, United States of America; 2 Pathology, University of South Alabama, Mobile, Alabama, United States of America; 3 Pharmacology & Center for Lung Biology, University of South Alabama, Mobile, Alabama, United States of America; 4 Cell Biology & Neuroscience, University of South Alabama, Mobile, Alabama, United States of America; 5 Pediatrics and Neonatology, University of South Alabama, Mobile, Alabama, United States of America; 6 Physiology, University of South Alabama, Mobile, Alabama, United States of America; International Centre for Genetic Engineering and Biotechnology, Italy

## Abstract

**Background:**

Adult mammalian cardiac myocytes are generally assumed to be terminally differentiated; nonetheless, a small fraction of cardiac myocytes have been shown to replicate during ventricular remodeling. However, the expression of Replication Factor C (RFC; RFC140/40/38/37/36) and DNA polymerase δ (Pol δ) proteins, which are required for DNA synthesis and cell proliferation, in the adult normal and hypertrophied hearts has been rarely studied.

**Methods:**

We performed qRT-PCR and Western blot analysis to determine the levels of RFC and Pol δ message and proteins in the adult normal cardiac myocytes and cardiac fibroblasts, as well as in adult normal and pulmonary arterial hypertension induced right ventricular hypertrophied hearts. Immunohistochemical analyses were performed to determine the localization of the re-expressed DNA replication and cell cycle proteins in adult normal (control) and hypertrophied right ventricle. We determined right ventricular cardiac myocyte polyploidy and chromosomal missegregation/aneuploidy using Fluorescent *in situ* hybridization (FISH) for rat chromosome 12.

**Results:**

RFC40-mRNA and protein was undetectable, whereas Pol δ message was detectable in the cardiac myocytes isolated from control adult hearts. Although RFC40 and Pol δ message and protein significantly increased in hypertrophied hearts as compared to the control hearts; however, this increase was marginal as compared to the fetal hearts. Immunohistochemical analyses revealed that in addition to RFC40, proliferative and mitotic markers such as cyclin A, phospho-Aurora A/B/C kinase and phospho-histone 3 were also re-expressed/up-regulated simultaneously in the cardiac myocytes. Interestingly, FISH analyses demonstrated cardiac myocytes polyploidy and chromosomal missegregation/aneuploidy in these hearts. Knock-down of endogenous RFC40 caused chromosomal missegregation/aneuploidy and decrease in the rat neonatal cardiac myocyte numbers.

**Conclusion:**

Our novel findings suggest that transcription of RFC40 is suppressed in the normal adult cardiac myocytes and its insufficient re-expression may be responsible for causing chromosomal missegregation/aneuploidy and in cardiac myocytes during right ventricular hypertrophy.

## Introduction

Adult mammalian heart is a terminally differentiated organ. It is made of two major cellular components, cardiac myocytes (CMs) and cardiac fibroblasts (CFs), which collectively constitute for approximately 90% of the cells in the myocardium [1]. CFs constitute for approximately 60–70% of the non-myocyte cells in the heart [1]. Although, they are known to retain their replicative properties in the adult heart, they are normally quiescent and proliferate into myofibroblasts only during patho-physiological remodeling of the heart [2]. In contrast, CMs which constitute about 30% of the human heart [1] cease to proliferate soon after birth and become post-mitotic or terminally differentiated [3], [4]. Therefore, the CMs in adult heart are unable to regenerate myocardial tissue after injury by ischemia-reperfusion insult and during heart failure. However, this paradigm has been shifted slightly in the past few years and there is a growing body of evidence that CMs from diseased heart can replicate during ventricular remodeling [5], [6]. Previous studies have demonstrated that transgenic over-expression of either oncogenes or cell cycle promoters leads to cell cycle activation in adult CMs [7]. Although, adult CMs have been shown to proliferate and regenerate following induction with growth factors [8], [9], nonetheless, clear evidence that normal adult CMs can undergo basal DNA synthesis is forthcoming [7]. Furthermore, the factor(s) responsible for preventing DNA replication in adult CMs is not clearly understood.

DNA replication is one of the challenging steps in the cell cycle and requires the collaboration of a formidable number of proteins [10]. In eukaryotes, several accessory proteins such as Replication Factor C (RFC) and Proliferating Cell Nuclear Antigen (PCNA), confer speed and high processivity to the replicative polymerases, DNA polymerases δ (Pol δ) and ε. The RFC functions as a clamp loader that loads PCNA, the clamp, onto DNA and consists of five subunits, RFC140, RFC40, RFC38, RFC37 and RFC36. The assembly of the RFC commits the cell to DNA replication and has significant influence on cell cycle transition from DNA replication to cell division [11]. Since CMs exit the cell cycle soon after birth, it is imperative to know the fate of these major DNA replication proteins in the adult heart cells. Although, previous studies have demonstrated down-regulation of DNA replication proteins in the adult heart [12], [13], [14], however, whether the expression of RFC and Pol δ proteins are down-regulated in adult normal CMs and CFs is still elusive. In this study, we show that transcription of RFC40 gene and translation of the catalytic subunit of Pol δ protein, p125, are suppressed in the normal adult CMs.

Considering the differences in the replicative properties of the CMs and CFs, post-natal growth of the heart is defined as CMs hypertrophy and CFs hyperplasia [2], subsequently leading to cardiac pathologies. Pulmonary arterial hypertension (PAH) is a fatal syndrome which is characterized by vasoconstriction and vascular remodeling of the pulmonary artery leading to increase in pulmonary arterial pressure [15]. Right ventricular (RV) hypertrophy occurs to compensate the severe pressure overload in PAH, thereby increasing the mortality rates in patients with PAH. Unlike left ventricular (LV) hypertrophy or failure, the molecular cause(s) in the pathogenesis of RV hypertrophy has rarely been studied and remains unknown. Besides, it is unclear whether increase in pulmonary arterial pressure, inhaled hypoxemia or combination of both triggers right heart growth in pulmonary hypertension diseases [16]. Several studies have demonstrated up-regulation of PCNA, in infracted and failing hearts [17] and right ventricular hypertrophy [18], and cell cycle proteins in acute myocardial infarction and heart failure [19], [20], however the status of RFC and Pol δ proteins in cardiac pathologies has rarely been studied. This study was undertaken to determine the expression of the RFC and Pol δ proteins in adult normal CMs and CFs and in PAH-induced RV hypertrophied hearts. In this study, we used an established Sugen-5416-induced PAH rat model, characterized by the formation of pulmonary vascular lesions closely resembling the one found in human PAH, leading to the development of severe RV hypertrophy and failure [21], [22]. We found that RFC40 and Pol δ message and protein significantly increased in hypertrophied hearts as compared to the control hearts along with re-expression of RFC40 and up-regulation of mitotic markers in the hypertrophied CMs. Fluorescent in situ hybridization studies for rat chromosome 12, demonstrated cardiac myocyte polyploidy and chromosomal missegregation/aneuploidy in the hypertrophied hearts, suggesting that re-expression of DNA replication proteins were in-sufficient to support cell proliferation. Consistent with this notion, we also observed chromosomal/aneuploidy missegregation and decreased cell numbers, following down-regulation of endogenous RFC40 in the rat neonatal CMs.

## Materials and Methods

Details of the following methods can be accessed in the Materials and Methods S1 file online.

### Animals

Pulmonary hypertension and cardiac hypertrophy was produced in adult male Sprague-Dawley rats (Harlan Laboratories, IN, USA) weighing 180 to 220 g (n = 10) as described previously [20] with vascular endothelial growth factor (VEGF) receptor blocker, Sugen-5416 (20 mg/kg) and exposed to hypoxia (10% O2) for 3 weeks (SUHx-3 wks). Five of these rats were then returned to normoxia (21% O2) for an additional 2 weeks (after Sugen-5416 injection +3 wks hypoxia; SUHxNx-5 wks) during which time they developed severe right ventricular hypertrophy. An additional 5 rats were used as time-matched normal controls (Control; without Sugen-5416 injection and hypoxia). Right and left ventricular systolic pressures were measured as previously described [22]. Right and left ventricles were separated from control and hypertrophied hearts for the experiments described in the present study. We studied two different time-points in the progression of hypertrophy; hypoxia plus pressure overload and pressure load alone.

Timed-pregnant female Sprague-Dawley rats were purchased from Charles River. The timed-pregnant dams were euthanized at the gestational age of day 15 and fetuses were immediately removed for isolation of their hearts. A pool of 50 whole hearts harvested from 15 day-old fetuses was used as a positive control. All experimental protocols were approved by the Institutional Animal Care and Use Committee of the University of South Alabama. The investigation conforms to the Guide for the Care and Use of Laboratory Animals published by the US National Institutes of Health. See Materials and Methods S1.

### Isolation of Ventricular Myocytes and Fibroblasts

The adult SD rats were deeply anesthetized by injection lethal dose of Nembutal (150 mg/kg) and single ventricular myocytes were isolated from rat hearts as described previously [23]. CFs were separated from the CMs by differential centrifugation and then used for either western blot analysis or t-RNA extraction. See Materials and Methods S1.

### Immunoblotting

CMs and CFs pellets, fetal hearts, LV and RV isolated from control, SUHx-3 wks and SUHxNx-5 wks treated heart tissues were homogenized in lysis buffer and the homogenates (50 µg) were then analyzed by 12% and 9% SDS-polyacrylamide gels. Western blot analysis was performed as described previously [10]. See Materials and Methods S1.

### Total RNA (t-RNA) Extraction and One-step Real-time RT-PCR

Total RNA was extracted from freshly isolated adult CMs, fetal, control and hypertrophic LV and RV tissues using the RNeasy fibrous tissue mini kit and from freshly isolated adult CFs using the RNeasy mini kit (Qiagen). Total RNA (50 ng) isolated from each of these samples (n = 5 for each group) was subjected to real-time one-step-RT-PCR using the iScript One-Step RT-PCR kit (Biorad). Assays for quantification of RFC40, p125, and GAPDH mRNA expression were conducted using the iCycler (BioRad). Amplified products for RFC40, p125, and GAPDH were visualized on 3% agarose gels. See Materials and Methods S11.

### Immunohistochemistry

Control and SuHxNx-5 wks RV slides were incubated with polyclonal anti-PCNA and anti-RFC40 (both from Abgent), anti-Cyclin A and anti-phospho-Histone 3 (Ser-10; pHis3; both from Santa Cruz Biotech) and anti-phospho-Aurora A/B/C kinase (Cell Signaling tech. Inc.) respectively, and monoclonal anti-cardiac Troponin-I (Santa Cruz Biotech) overnight at 4°C, and then with Alexa-568-labeled anti-rabbit and Alex-488-labeled anti-mouse secondary antibodies for one hour at room temperature. Nucleus was counterstained with DAPI and images were collected using an Olympus Plan x20/NA 0.25 Phi objective. See Materials and Methods S1.

Nuclear area was analyzed for >100 CM nuclei from three individual animals in control and hypertrophied RVs measurements using Image J software.

### DNA Probe and Labeling

BAC clone (Children’s Hospital Oakland Research Institute) representing the centromeric region of rat chromosome 12 (Cen12) was labeled with rhodamine (Cen12-ROX) by Empire Genomics.

### Fluorescent *in-situ* Hybridization (FISH) Analysis and Immunohistochemistry

Control and SuHxNx-5 wks RV slides were digested with Proteinase K (New England Biolabs, Inc.) and FISH was performed by co-hybridization of the tissues with the Cen12-ROX probe at 90°C for 10 min followed by overnight hybridization at 37°C. Nuclei were counterstained with 0.125 µg/ml of DAPI antifade (4′-6-diamidino-2-phenlindole; Cytocell). Slides were imaged with Spectral Imaging Software (Applied Spectral Imaging) using an Olympus BX61 microscope with 1000X magnification. Fifty CM nuclei were measured from three individual animals in control and hypertrophied RVs.

Immunohistochemical analysis was performed after imaging the slides for FISH signals as mentioned above with monoclonal anti-cardiac Troponin-I (1∶50) antibody. Nuclei were counterstained with DAPI. See Materials and Methods S1.

### DNA Replication Assays

M13mp18 DNA (1 pmole; New England Biolabs) was primed with 20 pmoles of a 80-mer-5′-biotinylated-M 13 primer (Invitrogen) as described previously [24], [25], [26], [27]. DNA synthesis in a standard reaction mixture (30 µl) containing 100 ng of primed M13mp18 was started by the addition of 20 µg of total protein lysates obtained from fetal, control-RV, SUHx-3 wks-RV and SUHxNx-5 wks-RV tissues, respectively, and the primer extension products were separated on urea (7 M) - polyacrylamide 6% (v/v) gel and visualized using the chemiluminescent nucleic acid detection kit (Pierce). See Materials and Methods S1.

### Immunodepletion Assays

Fetal hearts and RVs isolated from control, SUHx-3 wks and SUHxNx-5 wks hearts were homogenized as described above. 1 mg of total protein lysates from each sample was used for immunoprecipitation/immunodepletion experiments, using rabbit polyclonal anti-RFC40 antibody, as described previously [10]. 20 µg of total protein lysates obtained from each samples after immunodepletion were used for the DNA replication assay as mentioned above. See Materials and Methods S1.

### siRNA Studies

Rat Neonatal cardiac myocytes (RNCMs) were isolated as described previously [28] and grown in 12-well plates (0.5×10^6^ for western blot and cell number analyses) and 2-chambered slides (0.125×10^6^ for FISH) for 48 hr. RNCMs were then treated with non-targeting-siRNA (NT; 100 nM; Dharmacon) and On-Target plus smartpool RFC40-siRNA (100 nM; Dharmacon) respectively, for 72 hr. Cells lysates were subjected to Western blot analysis using anti-RFC40 antibody as described above. GAPDH was used as loading control. Before lysing, the RNCMs (n = 3) were trypsinized, resuspended in 1X PBS and counted using a hemocytometer. RNCMs grown in 2-chambered slides were subjected to FISH analysis following treatment with RFC40-siRNA as described previously [29], with few modifications (See Materials and Methods S1). Control and RFC40-siRNA-RNCM slides were co-hybridization with the Cen12-ROX probe (Red). Nuclei were counterstained with DAPI antifade (Blue). RFC40 knock-down was confirmed by performing immunohistochemical analyses for RFC40 (Green) in each sample.

### Statistical Analysis

Values are reported as mean±SE. ANOVA and *post-hoc* Fisher’s protected *t*-test or Student’s *t*-tests were used for analyzing all the data. Values of *P<*0.05 were considered significant. In all cases, the number of experimental determinations (*n*) was equal to the number of hearts from which LV & RV were harvested for this study.

## Results

### Differential Expression of the DNA Replication Proteins in CMs and CFs

**Figure 1:**
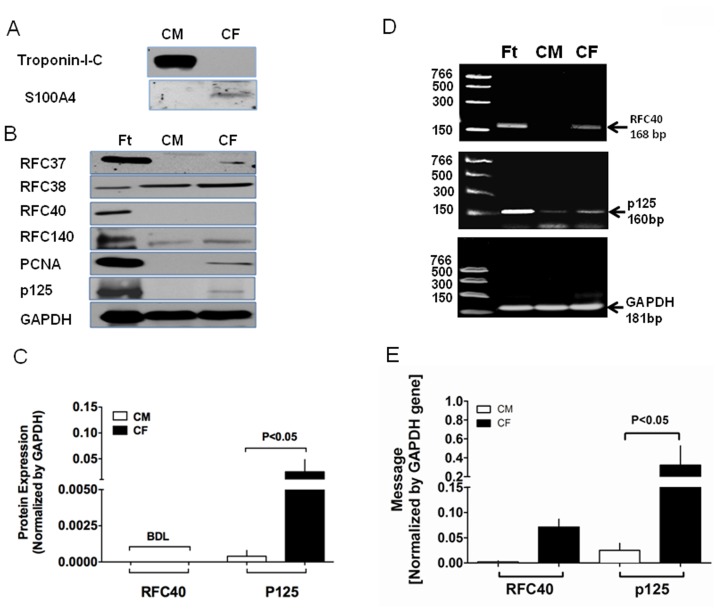
Differential expression of the DNA replication proteins in CMs and CFs. (**A**) CMs (n = 5) and CFs (n = 5) were isolated from normal adult heart and lysates (50 µg) were then analyzed by 12% or 9% SDS-polyacrylamide gels. Purity of the two fractions were confirmed by Western blot analyses for Troponin I-C (CMs) and S100A4 (CFs) proteins respectively. (**B**) Expression of RFC37, RFC38, RFC40, RFC140, PCNA and p125 proteins in the CMs and CFs was determined by Western blot analyses. Whole hearts isolated from 15 day-old fetus (n = 5) were used as a positive control (Ft). GAPDH was used as the loading control. (**C**) Graphs represent protein expression of RFC40 and p125, normalized by GAPDH in the CMs and fibroblasts, respectively. (**D**) Total RNA (50 ng) extracted from CMs and fibroblasts pellets (n = 5) was subjected to real-time one-step-RT-PCR. At the end of each run the amplified products of RFC40, p125 and GAPDH mRNA/cDNA were visualized on 3% agarose gels. Total RNA extracted from whole hearts of 15 day-old fetus (n = 5) were used as a positive control (Ft). (**E**) Graphs represent the mRNA levels in the CMs and CFs calculated from the crossing point deviation and normalized by GAPDH values. Values are mean ± SE. BDL: Below detectable levels.

We isolated CMs and CFs from normal adult heart and performed Western blot analyses for RFC, PCNA and p125 proteins to determine the cell type in which they are expressed. Purity of each fraction was first confirmed by cell specific markers, viz; Troponin I for CMs and S100A4 for CFs (Figure 1A). RFC40 (Figure 1B and C) protein was not detected in both CMs and CFs, while RFC140 and RFC38 proteins (Figure 1B) were expressed in both. Furthermore, PNCA (Figure 1B), RFC37 (Figure 1B) and p125 (Figure 1B and C) proteins were expressed only in the CFs. Real time qRT-PCR demonstrated that p125-mRNA was transcribed in both cell types (Figure 1D-middle panel), with significant higher expression in the CFs as compared to the CMs (Figure 1E). Strikingly, however, RFC40-mRNA was transcribed only in the CFs but not in the CMs (Figure 1D-top panel and 1 E).

### DNA Replication Proteins are Re-expressed in Hypertrophied Right Ventricle

**Figure 2:**
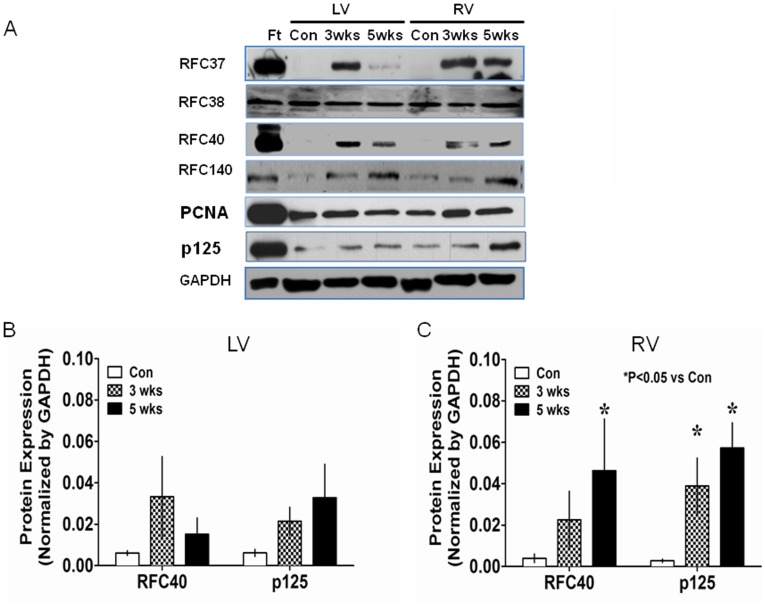
DNA replication proteins are re-expressed in hypertrophied RV. Total protein lysates (50 µg) obtained from LV and RV tissues (n = 5) of the control (Con), SUHx-3 wks (3 wks) and SUHxNx-5 wks (5 wks) were analyzed by 12% or 9% SDS-polyacrylamide gels. (**A**) Expression of RFC37, RFC38, RFC40, RFC140, PCNA and p125 proteins was determined by Western blot analyses. Whole hearts isolated from 15 day-old fetus (n = 5) were used as a positive control (Ft). GAPDH was used as the loading control. (**B and C**) Graphs represent summary data for the protein expression of RFC40 and p125 proteins in LV (B) and RV (C) normalized by GAPDH (n = 5). Values are mean ± SE. *indicates P<0.05 vs. control.[/LOOSEST]

Previous studies have demonstrated the up-regulation of fetal genes in most forms of hypertrophy [30]. Up-regulation of PCNA in the diseased hearts [17] raised the question of whether RFC37, RFC40 and p125 proteins, that are absent in the normal CMs, are also re-expressed/up-regulated in hypertrophied RV. We therefore used an established rat model of PAH-induced RV hypertrophy to determine the expression of all the DNA replication proteins. Adult rats injected subcutaneously with Sugen-5416 (SU; 20 mg/kg) and exposed to hypoxia (10% O2; SUHx) for 3 weeks (SUHx-3 wks; n = 5) and those that were returned to normoxia for an additional 2 weeks (SUHxNx-5 wks; n = 5) developed severe pulmonary hypertension as reflected by elevated RV systolic pressure (Figure S1 for the first supporting information figure). We performed Western blot analyses using the fetal, adult normal and hypertrophied hearts and found that the expression of p125, RFC37, RFC140 and PCNA were significantly down-regulated in adult control hearts as compared to fetal hearts (Figure 2A). Although, all these proteins were up-regulated in the hypertrophied hearts (Table 1), only p125 was significantly up-regulated both in the LV and RV of the hypertrophied hearts (Figure 2A, B and C). Interestingly, RFC40 expression was below detectable levels in the adult hearts, however, it was significantly re-expressed (P<0.05) in the hypertrophied RV (Figure 2C) than the LV (Figure 2B), although not at the fetal levels (Figure 2A).

**Table 1:** Expression of DNA replication proteins in control and hypertrophied hearts.

Protein	Control		SuHx-3 wks		SuHx-5 wks	
	LV	RV	LV	RV	LV	RV
RFC37	0.015 (0.02)	0.018 (0.03)	0.050^ ns^ (0.09)	0.045^ ns^ (0.07)	0.003^ ns^ (0.003)	0.023^ ns^ (0.04)
RCF38	0.096 (0.04)	0.158 (0.11)	0.060^ ns^ (0.06)	0.050^ ns^ (0.02)	0.080^ ns^ (0.04)	0.090^ ns^ (0.07)
RFC140	0.024 (0.02)	0.017 (0.01)	0.043^ ns^ (0.043)	0.045 * (0.031)	0.069^ ns^ (0.003)	0.031^ ns^ (0.03)
PCNA	0.055 (0.04)	0.028 (0.03)	0.125^ ns^ (0.11)	0.175^ ns^ (0.17)	0.070^ ns^ (0.03)	0.131 * (0.05)

All values are mean ±SD; ns-non-significant;

* P<0.05 vs control.

### Up-regulation/Re-expression of Replication Proteins in Hypertrophied Hearts Occurs at the Transcription Level

**Figure 3:**
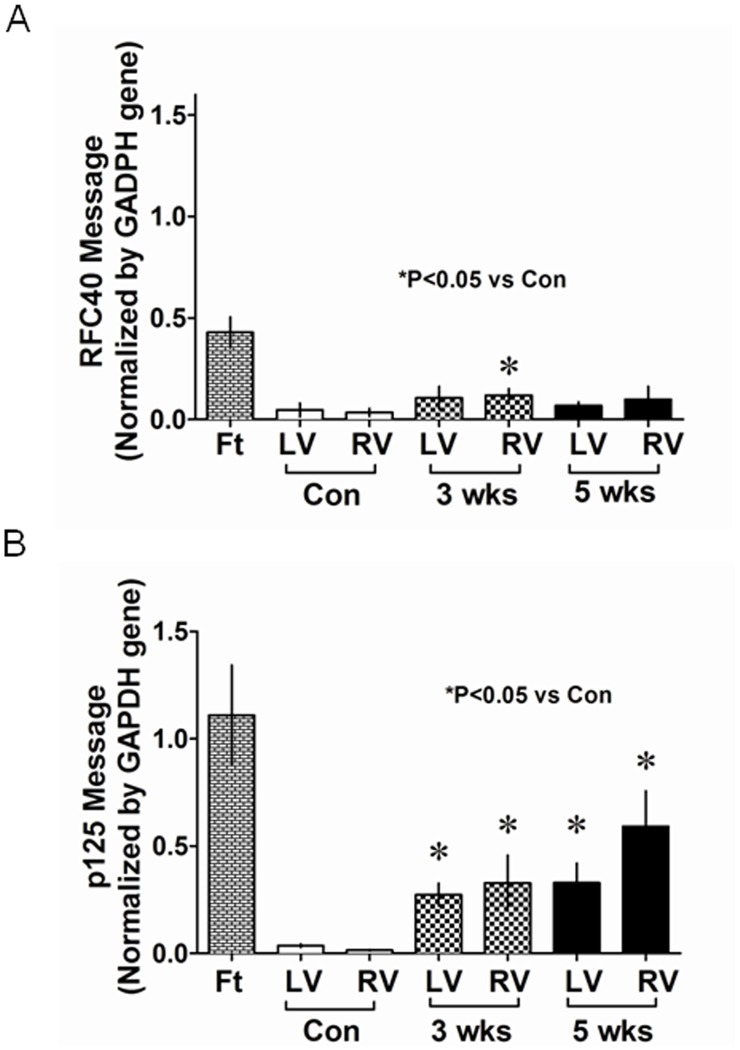
Up-regulation/re-expression of replication proteins in hypertrophied hearts occurs at the transcription level. Total RNA (50 ng) extracted from LV and RV tissues (n = 5) of the control (Con), SUHx-3 wks (3 wks) and SUHxNx-5 wks (5 wks) was subjected to real-time one-step-RT-PCR. The amplified products of RFC40, p125, and GAPDH mRNA/cDNA were visualized on 3% agarose gels at the end of each run. Total RNA extracted from whole hearts of 15 day-old fetus (n = 5) were used as a positive control (Ft). Graphs represent the changes in the mRNA levels for RFC40-mRNA (A) and p125-mRNA (B) calculated from the crossing point deviation of all the samples and normalized by GAPDH values. Values are mean ± SE. *indicates P<0.05 vs. control.

To determine whether the up-regulation/re-expression of the replication proteins in the hypertrophied myocardium occurred at the transcriptional level, we quantified mRNA for RFC40 and p125 in fetal, control and hypertrophied hearts by qRT-PCR and the amplified samples were ran on agarose gel to determine their molecular size and specificity at the end of each run (Figure S2 for the second supporting information figure). Quantitative analysis demonstrated that RFC40-mRNA and p125–mRNA in adult LV and RV decreased to 5–10% of those in fetal hearts (which may probably account for that present in the CFs). Interestingly, while RFC40-mRNA increased by 1.2 to 3.4-fold (P<0.05) in SUHx-3 wks and SUHxNx-5 wks hearts (Figure 3A), p125-mRNA increased by 7.6 to 40-fold in SUHx-3 wks and SUHxNx-5 wks hearts, respectively (Figure 3B) as compared to the control hearts. Nonetheless, this increase was marginal as compared to the fetal hearts.

### Immunohistochemical Analyses Revealed RFC40, PCNA, Cyclin A, Phospho-Aurora A/B/C Kinase and Phospho-Histone 3 Positive CMs Nuclei in Hypertrophied RV

**Figure 4:**
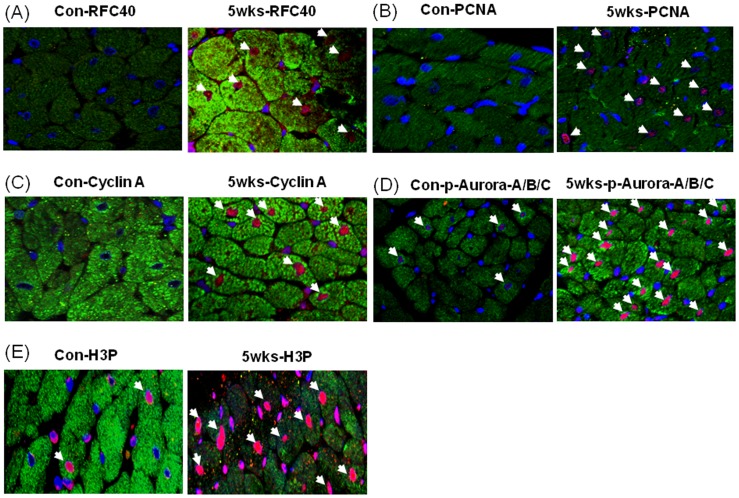
Immunohistochemical analyses revealed RFC40, PCNA, Cyclin A, phosphor-Aurora A/B/C kinase and pHis3 positive CMs nuclei in hypertrophied RV. Control (Con) and SUHxNx-5 wks (5 wks) RV sections (n = 3) were incubated with polyclonal anti-RFC40 (A) anti-PCNA (B), anti-Cyclin A (C), anti-phosho Aurora A/B/C kinase (p-Aurora-A/B/C; D) and anti-H3P (E) antibodies, respectively, and then with Alexa-568-labeled anti-rabbit and Alex-488-labeled anti-mouse secondary antibodies, respectively. Images were collected using an Olympus Plan x20/NA 0.25 Phi objective. In each experiment, all data were collected at identical imaging settings. Arrows indicates positive CMs.

To determine whether RFC40 was re-expressed in the hypertrophied heart CMs we performed immunohistochemical analyses for RFC40 in the RV isolated from the control and SUHxNx-5 wks treated hearts (since up-regulation of the replication proteins were more pronounced in the hypertrophied RVs). Although we found no staining for RFC40 in the control RV (Figure 4A; left), positive nuclear RFC40 staining (red/pink) of CMs was noted in the RV of the SUHxNx-5 wks treated hearts (Figure 4A; right). PCNA staining of the CMs were performed to demonstrate the proliferating status of the CMs. While no PCNA positive nuclei were found in control RV (Figure 4B; left), intense CMs staining was observed in SUHxNx-5 wks treated RV (Figure 4B; right). Furthermore, to determine if the CMs in the SUHxNx-5 wks treated hearts had re-entered the cell cycle, we performed immunohistochemical analyses for S-phase and G2/M phase proteins; Cyclin A, phospho-Aurora A (Thr288)/B (Thr232)/C (Thr198) kinase and phospho-Histone 3 (H3P; Ser-10) respectively, and observed no Cyclin A positive nuclei in the control RV (Figure 4C; left), but intense CMs staining for Cyclin A was observed in the SUHxNx-5 wks treated RV (Figure 4C; right). Although, we observed few phospho-Aurora A(Thr288)/B(Thr232)/C(Thr198) kinase (Figure 4D; left) and H3P (Figure 4E; left) positive nuclei in the control RV, the intensity of phospho-Aurora A(Thr288)/B(Thr232)/C(Thr198) kinase and H3P positive nuclei CMs significantly increased in the SUHxNx-5 wks treated RV (Figure 4D & E; right). Cardiac Troponin I was used as a cardiac myocyte marker to distinguish between the CMs and CFs staining.

### Cardiac Myocyte Polyploidy and Chromosomal Missegregation/aneuploidy was Observed in Hypertrophied RVs

**Figure 5:**
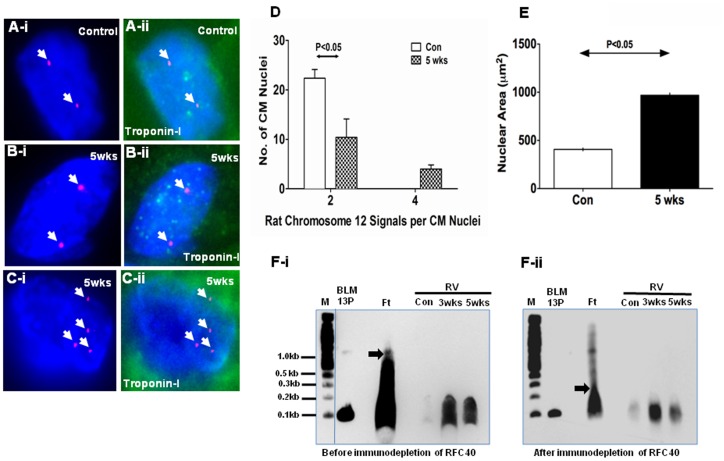
Cardiac myocyte polyploidy was observed in hypertrophied RVs. Control (Con) and SuHxNx-5 wks (5 wks) RV sections were used to perform FISH analyses by co-hybridization of the tissues with the Cen12-ROX probe (Red). Nuclei were counterstained with DAPI antifade (Blue) and slides were imaged with Spectral Imaging Software using an Olympus BX61 microscope with 1000X magnification. Two FISH signals for Cen12-ROX was observed in the CM nuclei from the control RV (A-i), whereas two (B-i) and four (C-i) signals were observed in the CM nuclei from the hypertrophied RVs. Localization of the CM nuclei in the control (A-ii) and hypertrophied (B-C-ii) RVs were confirmed by performing immunohistochemical analyses for cardiac Troponin I (Green). (D) Graph represents the number of FISH signals for Cen12-ROX observed per CM nuclei in the control and hypertrophied RVs. Fifty CM nuclei were measured from three individual animals in control and hypertrophied RVs. (E) Graph represents the nuclear area of >100 CM nuclei measured from three individual animals in control and hypertrophied RVs. Values are mean ± SE. *indicates P<0.05 vs. control. (F) In vitro DNA synthesis was carried out using primed M13mp18 ss DNA (100 ng) and protein lysates (20 µg) obtained from fetal, control-RV (Con), SUHx-3 wks-RV and SUHxNx-5 wks-RV tissues, respectively, before (F-i) and after (F-ii) immunodepletion of RFC40. The primer extension products were separated on a 7 M urea-6% (v/v) polyacrylamide gel and visualized by the chemiluminescent nucleic acid detection kit. Lane 1: Biotinylated 2-log DNA ladder (0.5 µg; M) in kilobases (B-lane 1; exposed for only 5 secs to visualize all the bands, is separated by a line). Lane 2: unannealed biotinylated M13 80-mer primer (BLM13P; 1 fmole). Blot is a representative of three such individual experiments.

To determine whether the increased expression of the DNA replication and S-phase proteins can support DNA synthesis during the pathogenesis of hypertrophy, we performed Fluorescent *in-situ* hybridization (FISH) on control and SUHxNx-5 wks-RVs, respectively. We used the BAC clone representing the bands 12p11–12q11 of rat chromosome 12 (Cen 12) labeled with 5-carboxyl-x-rhodamine (5-ROX; Cen12-ROX) as probe for FISH analyses (Figure S3 for the third supporting information figure). Interestingly, we noted two different observations in the CM nuclei of the hypertrophied RV- polyploidy and chromosomal missegregation/aneuploidy. **Polyploidy:** We observed two signals for Cen12-ROX (Red) in the CM nuclei from the control RV (Figure 5A-i). However, two (Figure 5B-i) and four (Figure 5C-i) signals in the CM nuclei from the hypertrophied RVs was observed. Localization of the CM nuclei in the control (Figure 5A-ii) and hypertrophied (Figure 5B–C-ii) RVs were confirmed by performing immunohistochemical analyses for cardiac Troponin I (Green). Furthermore, statistical analyses revealed that there was significant decrease (P<0.05) in the number of diploid CM nuclei and an increase in the number of polyploid nuclei (Figure 5D) with concomitant doubling in the nuclear area of the CM nuclei (Figure 5E) in the hypertrophied RVs as compared to the control RVs.

To substantiate the previous observations that RFC40 is vital for DNA replication, we performed *in-vitro* DNA replication assays using tissue homogenates obtained from fetal hearts, control-RV, SUHx-3 wks-RV and SUHxNx-5 wks-RV. Fetal heart lysate supported robust DNA replication as evident by the primer extension product obtained at ∼1.0 kb. Interestingly, the length of the products obtained with the hypertrophied heart lysates were ∼ 0.2 kb (Figure 5F-i), suggesting that these hearts partially regained their DNA replicative capabilities as compared to the control hearts. However, immunodepletion of RFC40 from the fetal heart and hypertrophied RV lysates reduced primer extension to less than 0.3 kb in the fetal heart and almost abolished it in the hypertrophied hearts (Figure 5F-ii), thus confirming the significance of RFC40 in DNA replication.

#### Chromosomal missegration/aneuploidy

**Figure 6:**
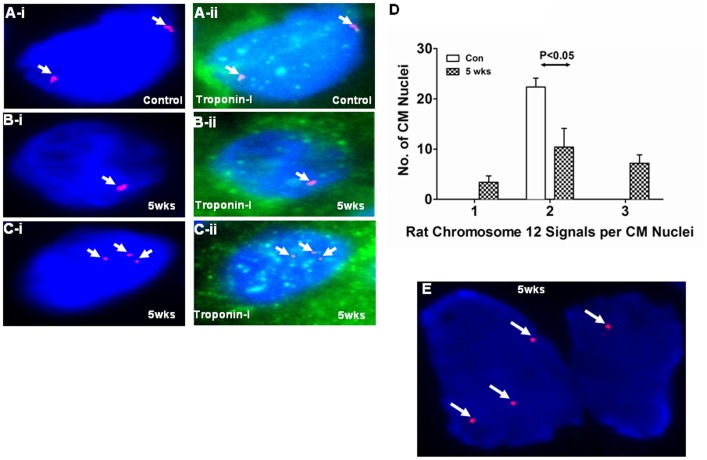
Chromosomal missegregation/Aneuploidy was observed in the CM nuclei from the hypertrophied RVs. Control (Con) and SuHxNx-5 wks (5 wks) RV sections were used to perform FISH analyses by co-hybridization of the tissues with the Cen12-ROX probe (Red). Nuclei were counterstained with DAPI antifade (Blue) and slides were imaged with Spectral Imaging Software using an Olympus BX61 microscope with 1000X magnification. Two FISH signals for Cen12-ROX was observed in the CM nuclei from the control RV (A-i), however, one (B-i) and three (C-i) signals were observed in the CM nuclei from the hypertrophied RVs. Localization of the CM nuclei in the control (A-ii) and hypertrophied (B–C-ii) RVs were confirmed by performing immunohistochemical analyses for cardiac Troponin I (Green). (D) Graph represents the number of FISH signals for Cen12-ROX observed per CM nuclei in the control and hypertrophied RVs. Fifty CM nuclei were measured from three individual animals in control and hypertrophied RVs. (E) Diving CM nuclei undergoing chromosomal missegregation in the hypertrophied RVs, with one nucleus receiving three sister chromatids while the other receiving only one chromatid.

In addition, to observing two and four signals for Cen12-ROX, we also observed one (Figure 6B-i), and three (Figure 6C-i) signals in several CM nuclei from the hypertrophied RVs. Localization of the CM nuclei in the control (Figure 6A-ii) and hypertrophied (Figure 6B–C-ii) RVs were confirmed by performing immunohistochemical analyses for cardiac Troponin I (Green). Furthermore, statistical analyses revealed that there was significant decrease (P<0.05) in the number of diploid CM nuclei and an increase in the number of aneuploid nuclei (Figure 6D). Additionally, we also observed some dividing CMs nuclei exhibiting chromosomal missegregation in the hypertrophied RVs with one nucleus receiving three sister chromatids of chromosome 12 while the other receiving only one sister chromatid (Figure 6E).

### Endogenous Knock-down of RFC40 in Rat Neonatal Cardiac Myocytes Results in Chromosomal Missegregation/aneuploidy

**Figure 7:**
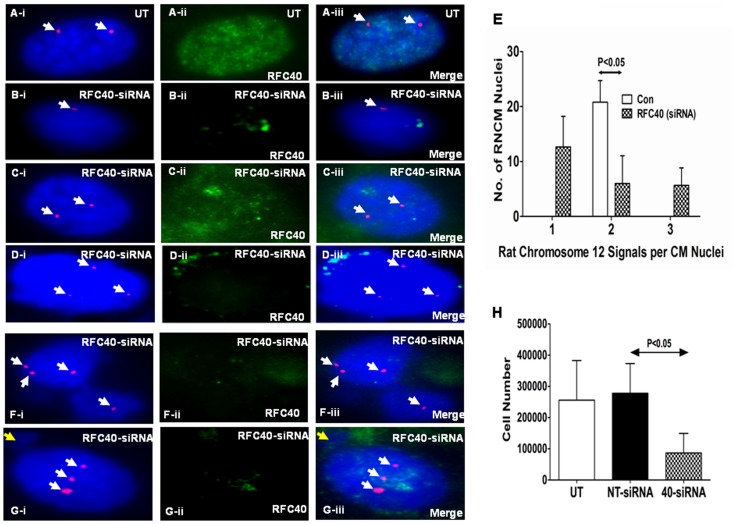
Endogenous knock-down of RFC40 in rat neonatal cardiac myocytes results in chromosomal missegregation/aneuploidy. Rat Neonatal cardiac myocytes (RNCMs) were isolated as described previously and grown in 12 well plates and 2-chambered slides for 48 hr. RNCMs were then treated with non-targeting-siRNA (NT) and On-Target plus smartpool RFC40-siRNA respectively, for 72 hr. (**A–D** and **F–G**) RNCMs grown in 2-chambered slides were subjected to FISH analysis following treatment with RFC40-siRNA. Untransfected (UT) and RFC40-siRNA-RNCM slides were co-hybridization with the Cen12-ROX probe (Red). Nuclei were counterstained with DAPI antifade (Blue). RFC40 knock-down was confirmed by performing immunohistochemical analyses for RFC40 (A–D-ii and F–G-ii; Green) in each sample. Merge images are shown in A–D-iii and F–G-iii. Panels A–D represents RNCM nuclei aneuploidy and panels F–G represent chromosomal missegregation. White arrows point to Cen12-ROX signals. Yellow arrow points to micronuclei in G-i & iii. (E) Graph represents the number of signals for Cen12-ROX observed per RNCM nuclei in the UT and RFC40-siRNA treated samples. Fifty RNCM nuclei in control and RFC40-siRNA treated samples from three individual experiments were measured. (H) Before lysing the cells grown in 12-well plates for western blot analyses (Fig. S4), the RNCMs (n = 3) were trypsinized, resuspended in 1X PBS and counted using a hemocytometer. Graph represents the number of RNCMs vs the different RNCM treated samples. Values are mean ± SE. * indicates P<0.05 vs. Untransfected (UT).

It has been previously demonstrated that RFC40 is required for accurate chromosomal segregation in *Drosophila* [31]. Since we observed significant aneuploidy and chromosomal missegregation in the CM nuclei, and since the re-expression of RFC40 in the hypertrophied RVs was less than 10% of that in the fetal hearts, we explored the possibility of whether insufficient re-expression of RFC40 must be responsible for aneuploidy and the chromosomal segregation defects observed in the hypertrophied hearts. To test this possibility, we knocked-down endogenous RFC40 protein in the neonatal (day 2) rat cardiac myocytes (RNCMs) using an On-Target plus smartpool RFC40-SiRNA for 72 hr. Western blot analysis demonstrated approximately 85% down-regulation of the RFC40 protein in the RFC40-siRNA treated samples as compared to untransfected (UT) and non-targeting-siRNA (NT) treated RNCMs (Figure S4 for the fourth supporting information figure). UT and RFC40-siRNA treated RNCMs were subjected to FISH analyses and RFC40 knock-down was confirmed by performing immunohistochemical analyses for RFC40 (Green) in each sample (Figure 7A–D-ii and F–G-ii). As expected, we observed only two signals for Cen12-ROX (Red) in the UT RNCM nuclei (Figure 7A-i). Interestingly, we observed one (Figure 7B-i) and three (Figure 7D-i) signals in the RFC40-siRNA treated RNCM nuclei. Although, we observed two signals (Figure 7C-i) for few RNCM nuclei treated with RFC40-siRNA, however, the RFC40 staining for these RNCM nuclei was stronger (Figure 7 C-ii) than that observed for the RFC40-siRNA treated RNCM nuclei with one (Figure 7B-ii) and three (Figure 7 D-ii) signals, respectively, suggesting that perhaps RFC40 was not knocked-down in these RNCMs (thus behaving as an internal control). Statistical analyses revealed significant increase in the number of aneuploid RNCMs (monosomy and trisomy for chromosome 12) following RFC40-siRNA treatment (Figure 7E). Furthermore, we also observed few bi-nucleated RNCMs with unequal chromosomal distribution (Figure 7F), similar to those observed in the hypertrophied RVs, and nuclei that exhibited both nondisjunction for Cen12-ROX (three signals) and micronuclei formation (Figure 7G). Moreover, cell number analyses demonstrated that RFC40-siRNA treated RNCMs significantly (P<0.05) decreased in cell numbers, as compared to Untransfected (UT) RNCMs (Figure 7H). Taken together, these data suggests that RFC40 is required for accurate chromosomal segregation as well as cell survival/proliferation in RNCMs.

## Discussion

Adult mammalian heart is a terminally differentiated organ. Although it is still controversial whether CMs proliferate in adult hearts, some studies have observed no cell cycle activity and only polyploidy in adult CMs [32], while other investigators believe that a small fraction (1–2%) of myocytes retain their ability to replicate throughout life [33]. However, regulation of DNA replication in CMs is not well understood and the status of the DNA replication machinery in adult normal and diseased CMs still remains elusive.

DNA replication in mammalian cell involves three major steps: (i) RFC assembles into a heteropentamer and loads PCNA onto the DNA, (ii) PNCA recruits Pol δ onto the DNA, and (iii) Pol δ ensures an error-free DNA synthesis and elongation prior to the cell division. Since the status of the DNA replication proteins has not been previously studied in the different cellular components of the adult heart, we investigated their expression in freshly isolated adult CMs and CFs. We found that RFC40 protein was not expressed in either the CMs or the CFs, whereas p125 protein, the catalytic subunit of Pol δ, was expressed only in the CFs. Furthermore, p125 mRNAs was present in both cell types. Interestingly, however, RFC40-mRNA was detectable only in CFs but not in CMs. These results suggest that transcription of RFC40 gene in normal adult heart is suppressed in the CMs which are terminally differentiated, while its translation is suppressed in the CFs. It implies that the cell regulates this vital DNA replication protein by turning its expression off at the gene level in the terminally differentiated cells.

Previous studies have demonstrated up-regulation of PCNA in infarcted and failing hearts [17], [18]. Similarly, cell cycle proteins have been shown to be up-regulated in human acute myocardial infarction and heart failure in dogs [19], [20]. However, there is a paucity of evidence demonstrating the status of core DNA replication proteins in adult normal or diseased heart muscle, during the pathogenesis of PAH-induced right ventricular hypertrophy. We, therefore, examined the expression of RFC and p125 proteins in a rat model of PAH in which pressure overload induces both RV hypertrophy and failure [21], [22]. We found that the expression of RFC40 protein was almost completely abolished in adult RV and LV as compared to the fetal heart, consistent with previous observations [14]. However, we demonstrate for the first time that RFC40 protein was re-expressed in the RVs of the hypertrophied hearts. Furthermore, although, RFC40 and p125 mRNA levels in the control heart decreased to 10% of that in the fetal hearts, their expression significantly increased (almost doubled for RFC40) in the hypertrophied RV as compared to control hearts, and that the up-regulation of these proteins occurred at the transcriptional level in hypertrophied hearts.

To determine the localization of the re-expressed DNA replication proteins in hypertrophied hearts, we stained the hypertrophied hearts (SUHxNX-5 wks) with RFC40 and found RFC40 positive CMs nuclei in the hypertrophied as compared to control RVs. Furthermore, to determine whether the CMs have re-entered the cell cycle in the hypertrophied heart, we stained the hypertrophied RV with a proliferative marker, PCNA, and observed PCNA positive CMs nuclei, thereby confirming previous observations [18]. Additionally, we also observed higher number of cyclin A (S-phase), and phospho-Aurora kinases and phospho-Histone 3 (G2/M-phase) positive CMs nuclei in the hypertrophied RVs as compared to control RVs, suggesting that the CMs could be proliferating in the PAH-induced hypertrophied RVs. Similarly, several studies have demonstrated significant up-regulation in phospho Histone 3 levels in LV hypertrophied cardiac myocytes [34], [35] and high mitotic index in various cardiac pathologies as compared to normal hearts [36].

To explore the possibility of whether re-expression of cell cycle and core DNA replication proteins can support DNA synthesis in the hypertrophied hearts, we performed FISH analyses on control and SUHxNx-5 wks RVs. While we mostly observed two FISH signals (diploid) for chromosome 12 in the CM nuclei from the control RV, the occurrence of two signals significantly decreased with frequent occurrence of four signals (tetraploid) and concomitant doubling in the nuclear area of the hypertrophied RV CM nuclei. These observations are consistent with previous findings wherein a increase in polyploidy and CM nuclear area was reported in human RV-pressure overload [37], spontaneously hypertensive rats [38] and myocardial infarct induced rat hearts [39]. These data implies that the CMs from the hypertrophied RVs were able to undergo one complete round of endoreplication through endomitosis characterized by duplication of the chromosomes without nuclear and cellular division along with the up-regulation of mitotic markers [40] such as Aurora kinases and H3P (Figure 4 D–E). These findings, however, are in striking contrast to that observed in left ventricular hypertrophy, where CMs of the hypertrophied LVs undergo several rounds of endoreplication [19], [41].

It has been shown that tetraploid cells demonstrate a higher degree of genomic instability than diploid cells and yield increased rates of chromosomal missegregation yielding two mononuclear cells with unequal chromosomal distribution that produce aneuploid cells in the subsequent round of division [42]. Consistently, in addition to diploid and tetraploid CM nuclei, we also observed significant CM nuclei with one and three FISH signals (Figure 6 B-i and C-i), suggesting the dominant presence of aneuploid CMs in the hypertrophied RVs. Similar defects in chromosomal segregation leading to aneuploidy have been reported in normal neural progenitor cells [43]; and fibroblasts [44] and brains [45], [46] derived from Alzheimer’s disease patients. Furthermore, it has been proposed that a single non-disjunction event could be tied with cleavage furrow regression resulting in the formation of one bi-nucleated cell with 3∶1 distribution of chromosomes [47]. Consistently, we found several dividing CM nuclei undergoing chromosomal missegregation (similar to those observed in mononuclear cells) with one nucleus receiving three sister chromatids while the other receiving only one sister chromatid.

Although, we found CMs polyploidy in the hypertrophied RVs, however, occurrence of defects in chromosomal segregation, in these same hearts, suggest that proteins involved in chromosomal segregation were not expressed to the fetal levels to support these processes. In lieu with this notion, it is noteworthy to mention that amongst all the DNA replication proteins studied in this article, RFC40 (whose expression is suppressed in the adult heart) is the only protein that is essential for DNA replication, DNA damage checkpoint response [11], maintenance of genomic stability [48], regulation of sister chromatid cohesion in mitosis and meiosis [49]–[50] and accurate chromosomal segregation in *Drosophila* [31], [49]. Consistently, immunodepletion of RFC40 from the fetal and hypertrophied RV tissue homogenates significantly decreased the in-vitro-DNA replication. Moreover, down-regulation of endogenous RFC40 in rat neonatal (day 2) CMs resulted in chromosomal segregation defects generating bi-nucleated RNCMs with abnormal chromosomal distribution, and aneuploid RNCMs along with micronuclei. Taken together, these data suggest that RFC40 is required for DNA replication as well as accurate chromosomal segregation in CMs.

Although, CM polyploidy has been previously reported in right and left ventricular hypertrophy [18], [37], [41], CMs still undergo apoptosis [51], [52] during the progression of hypertrophy. Consistently, a significantly increased rate of RV myocardial apoptosis has been demonstrated in the Sugen-5416-induced PAH rat model used in this study as compared to the control [16]. Interestingly, in addition to defects in chromosomal segregation, we also observed significant decrease in cell numbers, following down-regulation of endogenous RFC40 in the neonatal CMs. This data suggests that chromosomal missegregation/genomic instability in neonatal CMs and absence or loss of mitotic checkpoint must be responsible for causing cell death. We would, therefore, like to extrapolate our observations in the neonatal rat CMs to the hypertrophied heart rat models and speculate that chromosomal missegregation, that we observed in the hypertrophied hearts, may be responsible for causing CM death during the progression of hypertrophy. A fascinating comparison can be drawn from the adult neurons which are also known to be post-mitotic like the CMs [53]. It has been shown that neurons derived from Alzheimer’s disease patients re-enter cell cycle and undergo DNA replication. However, the frequency of tetraploid and aneuploid neurons are high in these patients [45], [46], predisposing them to cell death or apoptotic clearance [46], [53], [54]. Nonetheless, absence or down-regulation of other unknown factors influencing DNA replication, chromosomal segregation and cell cycle regulation in the adult CMs cannot be ruled out and warrants further investigation.

In conclusion, our novel findings suggest that transcription of RFC40 gene and translation of p125 protein is suppressed in the adult CMs. Furthermore, marginal re-expression (as compared to fetal hearts) of RFC40 protein, which is required for both DNA replication and chromosomal segregation, is not sufficient to support accurate chromosomal segregation in PAH-induced right ventricular hypertrophied CMS. Finally, absence of RFC40 protein in the adult heart due to transcriptional suppression provides potential explanation for the stalling of DNA replication in the adult heart.

## Supporting Information

Figure S1 Rats treated with VEGF receptor blocker and exposed to hypoxia developed RV and LV hypertrophy as shown in Figure 2.
(DOCX)

Figure S2 Visualization of qRT-PCR amplified products to determine their molecular size and specificity in the control and hypertrophied hearts as shown in Figure 3.
(DOCX)

Figure S3 FISH was first performed on rat blood to test the specificity and quality of the Cen12-ROX probe as shown in Figure 5 and 6.
(DOCX)

Figure S4 Western blot analyses to determine the efficiency of the RFC40-siRNA treatment in rat neonatal cardiac myocytes as shown in Figure 7.
(DOCX)

Materials and Methods S1 Detailed description of the Methods performed in this study.
(DOCX)
