# Supplementary material for: Down-Regulation of Replication Factor C-40 (RFC40) Causes Chromosomal Missegregation in Neonatal and Hypertrophic Adult Rat Cardiac Myocytes
Source: PLoS One. 2012 Jun 14;7(6):e39009. doi: 10.1371/journal.pone.0039009 (PMC3375256; doi:10.1371/journal.pone.0039009)
Supplement: Figure S1 — Rats treated with VEGF receptor blocker and exposed to hypoxia developed RV and LV hypertrophy as shown in Figure 2. (DOCX) [file pone.0039009.s001.docx]

**Figure S1. Rats treated with VEGF receptor blocker and exposed to hypoxia developed RV and LV hypertrophy.** Adult male Sprague-Dawley rats (n=10) were injected subcutaneously with Sugen-5416 (SU; 20 mg/kg) and exposed to hypoxia (10% O_2_) for 3 weeks (SUHx-3wks). Five of these rats were returned to normoxia (21% O_2_) for an additional 2 weeks (SUHxNx-5wks) and an additional 5 rats were used as normal control (Con). Prior to sacrifice, LV and RV systolic pressures were measured (**A**). The rat body weights (**B**), RV-to-LV + Septal (S) ratios (**C**), LV- and RV-to-body weight (normalized by body weight; **D**), total protein (normalized by wet ventricular weights; **E**) and protein-to-DNA ratios (**F**) in the LV and RV isolated from control and hypertrophied hearts (n=5 in each group) were estimated. Values are mean ± SE. * indicates P<0.05 vs. control.
