# Supplementary material for: Down-Regulation of Replication Factor C-40 (RFC40) Causes Chromosomal Missegregation in Neonatal and Hypertrophic Adult Rat Cardiac Myocytes
Source: PLoS One. 2012 Jun 14;7(6):e39009. doi: 10.1371/journal.pone.0039009 (PMC3375256; doi:10.1371/journal.pone.0039009)
Supplement: Figure S2 — Visualization of qRT-PCR amplified products to determine their molecular size and specificity in the control and hypertrophied hearts as shown in Figure 3. (DOCX) [file pone.0039009.s002.docx]

**Figure S2. Visualization of qRT-PCR amplified products to determine their molecular size and specificity in the control and hypertrophied hearts-** Total RNA (50 ng) extracted from LV and RV tissues (n=5) of the control (Con), SUHx-3wks (3wks) and SUHxNx-5wks (5wks) was subjected to real-time one-step-RT-PCR. The amplified products of RFC40, p125, and GAPDH mRNA/cDNA were visualized on 3% agarose gels at the end of each run. Total RNA extracted from whole hearts of 15 day-old fetus (n=5) were used as a positive control (Ft).
