# Supplementary material for: Down-Regulation of Replication Factor C-40 (RFC40) Causes Chromosomal Missegregation in Neonatal and Hypertrophic Adult Rat Cardiac Myocytes
Source: PLoS One. 2012 Jun 14;7(6):e39009. doi: 10.1371/journal.pone.0039009 (PMC3375256; doi:10.1371/journal.pone.0039009)
Supplement: Figure S3 — FISH was first performed on rat blood to test the specificity and quality of the Cen12-ROX probe as shown in Figure 5 and 6. (DOCX) [file pone.0039009.s003.docx]

**Figure S3. FISH was first performed on rat blood to test the specificity and quality of the Cen12-ROX probe-** Rat peripheral blood was collected, cultured, harvested and slides were dropped as described previously. FISH was performed according to the Vysis FISH WCP protocol. Briefly, the slides were incubated in 2X SSC at 37^o^C for 5 min and then hydrated through graded series of 70%, 85% and 100% ethanol for 2 min at room temperature. FISH was performed by co-hybridization of the tissues with the Cen12-ROX probe (Red) and denaturing the slides at 76^o^C for 4 min followed by overnight hybridization at 37^o^C. Post-hybridization washes for both blood and tissue slides were performed in 0.4X SSC plus 0.1% NP-40 followed by another wash with 2X SSC pus 0.5% NP-40. The nuclei were counterstained with 0.125 µg/ml of DAPI (Blue). Slides were imaged with Spectral Imaging Software using an Olympus BX61 microscope with 1000X magnification.
