# Supplementary material for: Down-Regulation of Replication Factor C-40 (RFC40) Causes Chromosomal Missegregation in Neonatal and Hypertrophic Adult Rat Cardiac Myocytes
Source: PLoS One. 2012 Jun 14;7(6):e39009. doi: 10.1371/journal.pone.0039009 (PMC3375256; doi:10.1371/journal.pone.0039009)
Supplement: Figure S4 — Western blot analyses to determine the efficiency of the RFC40-siRNA treatment in rat neonatal cardiac myocytes as shown in Figure 7. (DOCX) [file pone.0039009.s004.docx]

**Figure S4. Western blot analyses to determine the efficiency of the RFC4-siRNA treatment in rat neonatal cardiac myocytes-** Rat Neonatal cardiac myocytes (RNCMs) were isolated as described previously and grown in 12 well plates for 48 hr. RNCMs were then treated with non-targeting-siRNA (NT) and On-Target plus smartpool RFC40-siRNA respectively, for 72 hr. Cells lysates were subjected to Western blot analysis using anti-RFC40 antibody. GAPDH was used as loading control.
