# Supplementary material for: Down-Regulation of Replication Factor C-40 (RFC40) Causes Chromosomal Missegregation in Neonatal and Hypertrophic Adult Rat Cardiac Myocytes
Source: PLoS One. 2012 Jun 14;7(6):e39009. doi: 10.1371/journal.pone.0039009 (PMC3375256; doi:10.1371/journal.pone.0039009)
Supplement: Materials and Methods S1 — Detailed description of the Methods performed in this study. (DOCX) [file pone.0039009.s005.docx]

**Down-Regulation of Replication Factor C-40 (RFC40) causes chromosomal missegregation in neonatal and hypertrophic adult rat cardiac myocytes**

Hirotaka Ata,^a, #^ Deepa Shrestha,^b, #^ Masahiko Oka,^c, @^ Rikuo Ochi,^a^ Chian Ju Jong,^c^ Sarah Gebb,^d^ John Benjamin,^e^ Stephen Schaffer,^c^ Holly Hobart,^b^ James Downey,^f^ Ivan McMurtry,^b^ Rakhee Gupte. ^a,^*

**Supporting Information for Materials and Methods S1:**

**Animals**

To produce pulmonary hypertension and cardiac hypertrophy, adult male Sprague-Dawley rats (Harlan Laboratories) weighing 180 to 220 g (n=10) were injected subcutaneously with VEGF receptor blocker, Sugen-5416 (20 mg/kg) and exposed to hypoxia (10% O2) for 3 weeks (SUHx-3wks). Five of these rats were then returned to normoxia (21% O2) for an additional 2 weeks (after Sugen-5416 injection + 3 wks hypoxia; SUHxNx-5wks). An additional 5 rats were used as time-matched normal controls (Control; without Sugen-5416 injection and hypoxia). Prior to sacrifice, the control and pulmonary hypertensive rats were anesthetized with Nembutal (50mg/kg) and catheterized, and right and left ventricular systolic pressures were measured as previously described. ^30^ Rats were sacrificed by injecting lethal dose of Nembutal (150 mg/kg) and hearts were harvested. Left and right ventricles were separated from control and hypertrophied hearts for the experiments described in the present study (See Supplement Figure S1).

Timed-pregnant female Sprague-Dawley rats were purchased from Charles River. The timed-pregnant dams were euthanized at the gestational age of day 15 by an intra-peritoneal injection of 100 mg/kg of sodium pentobarbital suspended in isotonic saline. Fetuses were immediately removed, decapitated, and placed in ice cold phosphate buffered saline for isolation of their hearts. A pool of 50 whole hearts harvested from 15 day-old fetuses was used as a positive control.

**Isolation of ventricular myocytes and fibroblasts**

The adult SD rats were deeply anesthetized by injection of pentobarbitone sodium (40 mg/kg), and the hearts were excised and perfused using a Langendorff apparatus, first with normal Tyrode solution (135 mmol/L NaCl, 5.4 mmol/L KCl, 1.8 mmol/L CaCl_2_, 1.0 mmol/L MgCl_2_, 5 mmol/L HEPES, and 10 mmol/L glucose; pH was adjusted to 7.4) for a few minutes and then with nominally Ca^2+^-free Tyrode solution (adding 0.5 mmol/L EGTA to normal Tyrode solution) for 5 min, collagenase (Worthington Biochemical Corp.) solution for 25 min, and Kraft-Brühe (KB) solution ( 110 mmol/LKOH; 10 mmol/L taurine; 10 mmol/L oxalic acid; 70 mmol/L glutamic acid; 25 mmol/L KCl; 10 mmol/L KH_3_PO_4_; 5 mmol/L EGTA-Tris; 5 mmol/L Hepes-Tris; 10 mmol/L glucose; pH was adjusted 7.4) for 5 min. All solutions were saturated with 100% O_2_ and warmed to 37°C. After digestion, the ventricles were cut into small pieces with scissors, and cardiac myocytes were dissociated in KB solution in a shaking water bath and were dissociated by trituration with a fire-polished Pasteur pipette. KB solution was then filtered through a coarse metal sieve to obtain dissociated myocytes. After the cells were precipitated to the bottom of a beaker, they were aspirated into a Pasteur pipette, dispersed into normal Tyrode solution in another beaker, and incubated for 15 min at 37°C. The cell-containing beaker was removed from the bath and kept at room temperature.

The cardiac fibroblast/myocyte mixture was centrifuged at 565 rpm for 1.5 min and the supernatant containing the fibroblasts was collected in fresh tubes. The pelleted myocytes were washed with 1X PBS once and centrifuged at 565 rpm for 1.5 min. After discarding the supernatant, the myocytes pellets were used either for western blot analysis or t-RNA extraction. The supernatant containing the fibroblasts was centrifuged at 565 rpm for 1.5 min to remove any residual myocytes and then the supernatant was carefully transferred to fresh tube and centrifuged at 4000 rpm for 5 min. The pellet was washed once with 1X PBS and the resulting pellet was then passed through a 40µm cell strainer to obtain pure fibroblasts. After a final spin at 4000 rpm for 5 min the pellet was then used for either western blot analysis or t-RNA extraction. The purity of each fraction was confirmed using cardiac myocyte and fibroblast specific markers.

**Immunoblotting**

Fetal hearts, LV and RV isolated from control, SUHx-3wks and SUHxNx-5wks treated heart tissues were homogenized in lysis buffer (50 mmol/L Tris-HCl, pH 7.4, 150 mmol/L NaCl, 0.5% NP-40, 100 mmol/L phenylmethylsulfonyl fluoride (PMSF), 10 μg/ml aprotinin, 10 μg/ml leupeptin and 200 mmol/L pepstatin) and the homogenates (50 µg per sample) were then analyzed by 12% and 9% SDS-polyacrylamide gels. The primary antibodies used were: RFC140 (1:200), RFC38 (1:50), RFC37 (1:50), PCNA (1:200), Troponin I-C (1:200) all from Santa Cruz Biotech, Inc.; RFC40 (1:200; Abgent, Inc.); p125 (1:500, BD Bioscience), S100A4 (1: 100, Abcam), and GAPDH (1:1000, Cell Signaling Tech.). HRP-labeled secondary antibodies used were goat anti-rabbit, goat anti-mouse, bovine anti-goat and goat anti-rat (Santa Cruz). Specific proteins were detected by chemiluminescence (West Pico; Pierce). RFC36 antibodies from Santa Cruz and Abgent were not able to conclusively detect rat RFC36 and hence the data is not included in this manuscript.

**Protein-to-DNA ratio**

Fetal, control and hypertrophied LV and RV tissues (15-20 mg, n =5 for each group) were homogenized in 180 µl of ATL buffer and used for the estimation of protein to DNA ratio using the QIAamp DNA mini kit (Qiagen) as per the manufacturer’s protocol.

**Total RNA (t-RNA) extraction and One-step real-time RT-PCR**

Total RNA was extracted from fetal, control and hypertrophic LV and RV tissues and freshly isolated adult cardiac myocytes using the RNeasy fibrous tissue mini kit and from freshly isolated adult cardiac fibroblasts using the RNeasy mini kit (Qiagen). Total RNA (50 ng) isolated from each of these samples (n=5 for each group) was subjected to real-time one-step-RT-PCR using the iScript One-Step RT-PCR kit (Biorad). Assays for quantification of RFC40, p125, and GAPDH mRNA expression were conducted on the iCycler (BioRad) using specific primers (all primers were purchased from Invitrogen) as follows: (i) RFC40 (168bp): forward,5’atggaggttcaggagagcggc3’ and reverse, 5' CTCCAGCCTGCTcacagtgtcttc 3' ; (ii) p125 (160 bp): forward, 5' GAGTACAAGCTCCGCTCCTACACGC3' and reverse, 5’GCAGAAAGGCATCCTTCAGGCAG3’; (iii) GAPDH (181 bp): forward, 5' CTCATGACCACAGTCCATGCCATC3' and reverse, 5' CGGAAGGCCATGCCAGTGAG 3'. RFC40, p125 and GAPDH mRNA/cDNA amplification was programmed at 55°C for 10 min for cDNA synthesis followed by 95°C for 5 min (RT enzyme inactivation), and 40 cycles of 95°C for 10 s, 60°C for 30 s, and 72°C for 30s (data collection point). Melting curve analysis was subsequently conducted in order to verify the purity of the products. The fold increase in the mRNA levels were calculated from the crossing point (Ct) deviation of all the samples and normalized with GAPDH values. Amplified products were visualized on 4% agarose gels (See Supplement Figure S2). Rat RFC37 and RFC36 mRNA sequences were posted in NCBI-Genbank, during the preparation of this manuscript (July 16, 2011), however final review for these sequences has not being completed to date.

**Immunohistochemistry**

Control and SuHxNx-5wks RV were embedded in paraffin and cut into 5-7 µm sections. Slides were incubated at 60^o^C for 30 min prior to de-paraffinization followed by three 5 min incubations with xylene and two washes each in 100% ethanol, 95% ethanol and distilled water, respectively. Antigen retrieval was performed with 1X citrate buffer at 100^o^C for 20 min and blocking was carried out in 5% normal goat serum (Sigma) at room temperature for one hour. Subsequently, the sections were incubated overnight at 4^o^C with two antibodies per slide. All slides were incubated with monoclonal anti-cardiac Troponin-I (1:100; Santa Cruz Biotech) antibody and either polyclonal anti-PCNA (1:20; Abgent), anti-RFC40 (1:200; Abgent), Cyclin A (1:200; Santa Cruz Biotech), phospho-Aurora A (Thr288)/B (Thr232)/C (Thr198) kinase (Cell Signaling Technology) or phospho-Histone 3 (Ser-10; 1:200; Santa Cruz Biotech) antibodies respectively. Secondary antibodies used were Alexa-568-labeled anti-rabbit and Alexa-488-labeled anti-mouse antibodies for one hour at room temperature. Nuclei were counterstained with DAPI. Images of the stained sections were collected using an Olympus Plan x20/NA 0.25 Phi objective. In each experiment, all data were collected at identical imaging settings.

**DNA probe and labeling**

A BAC clone (Children’s Hospital Oakland Research Institute) representing the bands 12p11-12q11 of rat chromosome 12 (Cen 12; 12563307-12814760) was labeled with 5-carboxyl-x-rhodamine (5-ROX) by Empire Genomics.

**Fluorescent in-situ hybridization (FISH) analysis**

FISH was first performed on rat blood to test the specificity and quality of the Cen12-ROX probe. Peripheral blood was collected, cultured, harvested and slides were dropped as described previously [S1, S2]. FISH was performed according to the Vysis FISH WCP protocol. Briefly, the slides were incubated in 2X SSC (20X SSC: 3 M NaCl, 0.3 M Sodium Citrate; pH=7.0; Promega) at 37^o^C for 5 min and then hydrated through graded series of 70%, 85% and 100% ethanol for 2 min at room temperature. FISH was performed by co-hybridization of the tissues with the Cen12-ROX probe (1 µl + 9 µl hybridization buffer from Empire Genomics) and denaturing the slides at 76^o^C for 4 min followed by overnight hybridization at 37^o^C. (See Supplement Figure S3). The protocol for FISH using heart tissues and rat neonatal cardiac myocytes was as follows: **(a) Heart Tissues:** Control and SuHxNx-5wks RV were embedded in paraffin and cut into 5-7 µm sections. The slides were de-paraffinized with xylene and xylene/ethanol (1:1), hydrated with 100% ethanol and digested with Proteinase K (New England Biolabs) for 25 min at 37^o^C. The slides were then incubated with denaturation buffer (70% formamide in 2xSSC; pH=7.5) at 73^o^C for 5 min and then hydrated through graded series of 70%, 85% and 100% ethanol [S3]. FISH was performed by co-hybridization of the tissues with the Cen12-ROX probe (2 µl + 8 µl hybridization buffer) and denaturing the slides at 90^o^C for 10 min followed by overnight hybridization at 37^o^C. Post-hybridization washes for both blood and tissue slides were performed in 0.4X SSC plus 0.1% NP-40 followed by another wash with 2X SSC pus 0.5% NP-40. The nuclei were counterstained with 0.125 µg/ml of DAPI (4’-6-diamidino-2-phenlindole; Cytocell, Cambridge, UK). Slides were imaged with Spectral Imaging Software (Applied Spectral Imaging) using an Olympus BX61 microscope with 1000X magnification. **(b) Rat neonatal cardiac myocytes:** Rat Neonatal cardiac myocytes (RNCM) were isolated as described previously and grown (0.125x10^6^) in 2-chambered slides for 48 hr. RNCM were then treated with On-Target plus smartpool RFC40-siRNA (100 nM; Dharmacon) using of Dharmafect Reagent-1 for 72 hr and subjected to FISH analysis. The RNCM were fixed with 96% ethanol for 15 min at room temperature, bath in 0.075 M KCl for 12 min at 37^o^C and fixed again with methanol:acetic acid (3:1) for 10 min at room temperature [29]. Slides were then incubated in 2X SSC for 2 min at 37^o^C and then hydrated through graded series of 75%, 85% and 100% ethanol for 2 min at room temperature. FISH was performed by co-hybridization of the tissues with the Cen12-ROX probe (2 µl + 8 µl hybridization buffer) and denaturing the slides at 76^o^C for 4 min followed by overnight hybridization at 37^o^C.

Immunohistochemical analysis was performed after imaging the slides for FISH signals as mentioned above. The slides were blocked for an hour at room temperature followed by incubated with monoclonal anti-cardiac Troponin-I (1:50-for tissue) and polyclonal anti-RFC40 (1:200-for RNCM) antibodies respectively at 37^o^C for an hour. Following incubation with Alexa-488-labeled anti-mouse (for Troponin I-C) and Alexa-488-anti-rabbit (for RFC40) secondary antibodies respectively, for one hour at room temperature, the nuclei were counterstained with DAPI.

**DNA replication assays**

M13mp18 ss DNA was obtained from New England Biolabs. A 80-mer M13primer (5’-GCTGATAAATTAATGCCGGAGAGGGTAGCTATTTTTGAGAGATCTACAAAGGCTATCAGGTCATTGCCTGAGAGTCTGGA-3’) that was complementary to nucleotides 6899-6978 of the M13mp18 DNA was 5’-biotinylated as described previously, and PAGE purified by Invitrogen. M13mp18 DNA (1pmole) was primed with 20 pmoles of biotin-labeled M13 primer in 10 mmol/L Tris-HCl (pH 7.4), 1 mmol/L EDTA and 100 mmol/L NaCl. The 50 µl reaction mixtures were heated at 95^o^C for 1 min and cooled slowly to room temperature for 2 hr. The annealed structure was purified away from the unannealed biotin-labeled M13 primer on a 1% agarose gel using the GeneClean II kit (MP Biomedicals). The standard reaction mixture (30 µl) for the DNA replication assay contained 100 ng of primed M13mp18 DNA, 30 mmol/L Hepes (pH 8.0), 7 mmol/L MgCl_2_, 40 mmol/L NaCl, 0.5 mmol/L dithiothreitol, 0.1 mg/ml bovine serum albumin and 0.1mmol/L dNTP. The reaction mixtures were pre-incubated at 0^o^C for 10 min. DNA synthesis was started by the addition of 20 µg of total protein lysates obtained from fetal, control-RV, SUHx-3wks-RV and SUHxNx-5wks-RV tissues, respectively, as described previously and incubated at 37^o^C for 60 min. The reaction was terminated by incubating the reactions mixtures with stop buffer (25 µl; 20 mmol/L EDTA, 0.5% SDS and 0.2 mg/ml of Proteinase K) at 37^o^C for 30 min. The reaction mixtures (25 µl) were mixed with 5 µl of denaturing loading buffer (0.3 M Tris-HCl, 5% SDS, 50% glycerol, 100 mmol/L dithiothreitol, 0.025% xylene cyanol and 0.025% bromophenol blue) and the primer extension products were separated on urea (7 M)- polyacrylamide 6% (v/v) gel. After transferring the gel onto a positively charged nylon membrane (Hybond-XL; Amersham Pharmacia), the biotinylated primer extension products were incubated with a streptavidin-HRP conjugate as described previously, and visualized using the chemiluminescent nucleic acid detection kit (Pierce). Biotinylated 2-log DNA ladder (0.5µg; New England Biolabs) was used as a molecular size marker.

**Immunodepletion Assays**

Fetal hearts and RVs isolated from control, SUHx-3wks and SUHxNx-5wks hearts were homogenized as described above. 1 mg of total protein lysates from each sample was used for immunoprecipitation experiments, using rabbit polyclonal anti-RFC40 antibody, as described previously. The immunocomplexes were incubated with A and G- congujated-agarose beads (Santa Cruz) at 4^o^C overnight and spun at 13, 2000 rpm for 2 min. The supernatant was transferred to fresh tubes and protein estimation of the immunodepleted samples was performed as mentioned above. 20 µg of total protein lysates from each samples were used for the DNA replication assay as mentioned above.

**siRNA studies**

Rat Neonatal cardiac myocytes (RNCMs) were isolated as described previously [28] and grown in 12-well plates (0.5 x10^6^) for 48 hr. RNCMs were then treated with non-targeting-siRNA (NT; 100 nM; Dharmacon) and On-Target plus smartpool RFC40-siRNA (100 nM; Dharmacon) respectively, for 72 hr. Cells lysates were subjected to Western blot analysis using anti-RFC40 antibody as described above. GAPDH was used as loading control (See Supplement Figure S4).

References:

1. Hack MS, Lawce HJ (1980) The Association of Cytogenetic Technologists Laboratory Manual. pp. 70-77.
2. Barch ,MJ (1991) The Association of Cytogenetic Technologists Laboratory Manual. pp. 24-30.
3. Henegariu O, Fluorescent in situ hybridization guide and troubleshooting.

Available :<http://info.med.yale.edu/genetics/ward/tavi/> Accessed: 2011 Aug 08.
